# Grasping Discriminates between Object Sizes Less Not More Accurately than the Perceptual System

**DOI:** 10.3390/vision3030036

**Published:** 2019-07-19

**Authors:** Frederic Göhringer, Miriam Löhr-Limpens, Constanze Hesse, Thomas Schenk

**Affiliations:** 1 Lehrstuhl für Klinische Neuropsychologie, Ludwig-Maximilian University Munich, Leopoldstr. 13, 80802 Munich, Germany; 2 School of Psychology, University of Aberdeen King’s College, William Guild Building, Aberdeen AB24 3FX, UK

**Keywords:** perception-action model, Two Visual Streams Hypothesis, grasping, object size, Just Noticeable Difference

## Abstract

Ganel, Freud, Chajut, and Algom (2012) demonstrated that maximum grip apertures (MGAs) differ significantly when grasping perceptually identical objects. From this finding they concluded that the visual size information used by the motor system is more accurate than the visual size information available to the perceptual system. A direct comparison between the accuracy in the perception and the action system is, however, problematic, given that accuracy in the perceptual task is measured using a dichotomous variable, while accuracy in the visuomotor task is determined using a continuous variable. We addressed this problem by dichotomizing the visuomotor measures. Using this approach, our results show that size discrimination in grasping is in fact inferior to perceptual discrimination therefore contradicting the original suggestion put forward by Ganel and colleagues.

## 1. Introduction

According to the Perception-Action Model (PAM), suggested by Milner and Goodale [1,2], the visual system consists of two functionally separated streams, the dorsal stream and the ventral stream. The ventral stream provides vision for perception and the dorsal stream provides vision for action. The model was first formulated to account for deficits observed in patients suffering from ventral or dorsal stream damage. Visual form agnosic patient D.F., who suffered from ventral stream lesions, was found to still have functioning motor control, despite her severely impaired visual perception [3,4]. In contrast, optic ataxia patients suffering from dorsal lesions tend to show impaired motor control, while their visual perception remains largely normal. [5].

The model contains an important assertion. The visual processes taking place in the two distinct streams use different representations and different processing modes [6]. In principle, it is therefore possible to test this two-visual pathway hypothesis also in healthy participants. For example, finding that some processing error (or to put it more neutrally: processing feature) affects only perceptual tasks, but not visuomotor tasks, could be taken as an indication that the two tasks use different visual representations and that only one type of representation is affected by this error. In this context, the most extensively studied error is the susceptibility to perceptual illusions. Many studies have suggested that perceptual illusions affect perceptual but not visuomotor tasks (e.g., [7,8]). However, this evidence has been challenged in numerous studies, and counter-examples and alternative accounts have been provided (for reviews, see Carey [9], Bruno [10], Franz [11], Franz and Gegenfurtner [12], Bruno and Franz [13], Schenk, et al. [14], and Schenk [15]). A recent large-scale, multicenter, preregistered study showed that for the Ebbinghaus illusion, one of the most commonly studied illusions, the illusion effects are pretty much identical for perception and action [16,17]. Illusions are, however, not the only tool employed to demonstrate the distinctness of representations in the perceptual and the visuomotor system. Ganel and Goodale [18], for example, showed that the Garner-interference effect influences perceptual size-judgements but not the size of the grip apertures in a visuomotor task. Furthermore, Ganel, et al. [19] also reported that a fundamental psychophysical law, Weber’s law, is selectively violated in grasping. Finally, Singhal et al. [20] showed that a more general cognitive phenomenon, namely the finding that the concurrent execution of two tasks creates performance costs for at least one of the two tasks, reliably occurs in tasks that can be assigned to the perceptual system but is much less prominent in tasks assigned to the visuomotor system. However, all these approaches have been met with counter-evidence and are currently bogged down in controversy [21,22,23,24,25,26,27,28,29].

In 2012, Ganel, Freud, Chajut, and Algom [30] proposed another novel approach to test for the existence of distinct processing modes in perception and action. They presented participants with objects that differed in size by only 0.5 mm. Participants were first asked to indicate verbally which of the two objects was the bigger one, and subsequently had to grasp the object directly in front of them. It turned out that participants were at chance level with their verbal judgements. Yet, when their hand-openings during the grasping movements were analyzed, those hand-openings differed significantly for the smaller and bigger objects. Most interestingly, even when observers erroneously labelled the bigger object as the smaller one their hand-opening was still (on average) bigger than when they erroneously labelled the smaller object as being bigger. Thus, it seemed that observers’ hand-openings were not affected by their conscious size judgement. Their hands reliably adjusted to the true physical size of the objects, even when they could not perceptually discriminate between those objects. Based on these findings, Ganel and colleagues concluded that perceptual judgement and grasping are based on distinct representations of visual size and that the size representation for grasping is more precise than the one used for explicit perceptual judgements. Furthermore, these findings were interpreted as support for Milner and Goodale’s claim that vision for perception and vision for action are served by distinct neural pathways.

This conclusion relies, however, on the assumption that both tasks use visual size as their main input which has been challenged by Smeets and Brenner [31]. They presented a model which could correctly account for most aspects of grasping movements, while assuming that the sensorimotor system does not compute object size but instead determines the optimal contact positions for the grasping digits (typically index finger and thumb) on the target object. They demonstrated that using this assumption, grip apertures still positively correlate with object size, despite this parameter never being explicitly computed. On the basis of this account, it would not be expected that visual requirements for perceptual size-discrimination and grasping are identical and thus, in the context of this model, it is hardly newsworthy that significant differences can be found when grasping objects whose sizes cannot be reliably discriminated.

While we accept the more general point, namely that grasping and size discrimination do not necessarily use the same sensory inputs and that grasping should not be treated as the motor equivalent of a size-judgement task (for a more detailed discussion of this point, see Hesse et al. [32] and Schenk et al. [24]), we do think that there is evidence to suggest that visual size information is commonly used for grasping in healthy participants (albeit possibly not, or to a lesser extent, by patients with agnosia [33,34]). For example, the above discussed finding that grasping and perceptual judgements are impacted very similarly by visual size illusions [16] seems to indicate that object size is used also for grasping (for a slightly different view, see de Grave, et al. [35]). Further evidence comes from studies on grasping familiar objects. For example, McIntosh and Lashley [36] showed that the assumptions that we make about the size of familiar objects have a significant impact on how we grasp those objects. Taken together these findings suggest that size does play an important role in shaping our grasping response. Thus, if we accept that grasping relies on object size information, it is indeed surprising and noteworthy that in grasping we seem to be able to distinguish between object sizes that are perceptually identical. However, while we do not question the assumption that both grasping and perceptual judgement rely on object size information, we challenge the claim by Ganel, Freud, Chajut, and Algom [30] that there is a dissociation in the accuracy of this information.

So, let us have a closer look at the evidence upon which the claim is based that the representation of size underlying grasping is superior to the representation underlying perceptual judgement. On average observers guessed the correct size of the object only in 58.7% of trials, i.e., barely above chance. In contrast, when the average maximum grip aperture (MGA) was analyzed a reliable and significant difference between the MGAs for the smaller and bigger object emerged. However, is this contrast enough to claim that the hand distinguishes between objects more reliably than the observer? To illustrate the problem with this claim, we can take the example of body height in Scottish and English men. The mean height of adult male Scots is 176 cm, and thus approximately 2 cm less than the mean height of English adult males. Thus, if we took a representative sample of Scottish and English males to compare their average height, we would expect to find that the average Scottish height is significantly below that of the English sample. Nevertheless, would we be asked to assign nationality on the basis of body height we would make frequent errors. The same analogy holds for comparing grasping and perceptual data. MGAs for smaller objects may well be significantly smaller than for bigger objects, but chances are there are many grasping responses directed to the bigger object producing smaller MGAs than those found for grasping responses directed to the smaller object. Thus, the following question arises: if we tried to guess the size of the target on the basis of the observed MGAs, would the number of correct guesses significantly exceed the number of correct guesses achieved by the observers in the perceptual judgement task? To address this question, we replicated the study by Ganel, Freud, Chajut, and Algom [30] and re-analyzed the findings by obtaining measures for size-classification accuracy based on the MGAs of the participants grasping responses.

In total, we performed three experiments. In the first experiment, we aimed to replicate the first experiment from Ganel, Freud, Chajut, and Algom [30]. Surprisingly, our findings differed from those obtained by Ganel, Freud, Chajut, and Algom [30] already prior to the proposed re-analysis of the data. We therefore decided to replicate this experiment (Experiment 2) with a new sample of participants to check whether our original findings were reliable. The second experiment produced a new pattern of findings which (again prior to the proposed re-analysis) were more similar to the results obtained by Ganel and colleagues [30]. In our final experiment (Experiment 3) we examined the role of hand-sight and asked participants to perform the tasks used in Experiments 1 and 2 once under closed-loop conditions (i.e., moving hand remained visible throughout the trial) and again under open-loop conditions (i.e., hand only visible at the start of the movement). In this last experiment we found for the closed loop condition a pattern more similar to Experiment 1.

## 2. Materials and Methods

### 2.1. Experimental Setup

**Figure 1:**
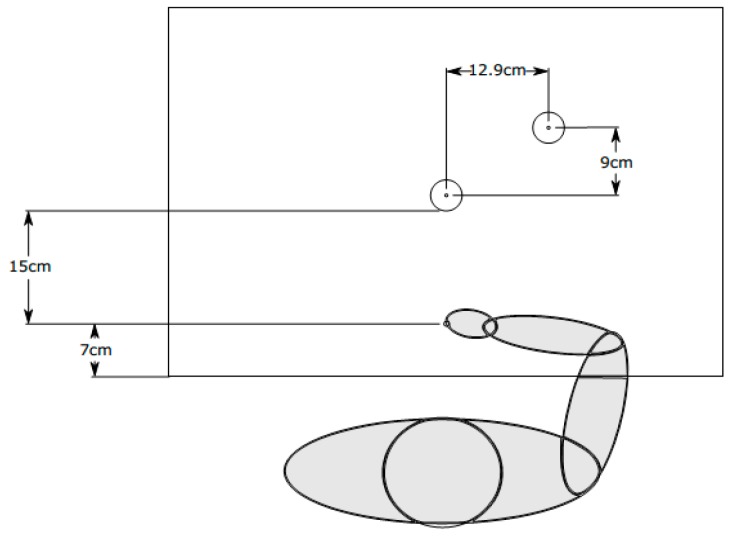
**Experimental setup**. Shown here is the stimulus arrangement of one exemplary trial. At the beginning of each trial, participants grasped the starting pole. Participants were then asked to grasp the object positioned straight ahead, seen here as the larger disk. The second disk was always positioned either to the right and back, as shown here, or to the left and back. Participants then had to indicate whether the object in front was larger or smaller than the object in the back. In half of the blocks the order was reversed with participants first indicating the size and then grasping the disk.

We followed Ganel, Freud, Chajut, and Algom [30] in the design of the study. The objects we used had the same sizes of 40 mm and 40.5 mm and a height of 2 mm. The target object was placed 15 cm in front of the starting position of the participant’s hand in Experiment 1 and 2, and 8 cm in front of the starting position in Experiment 3. The second object was always placed at a horizontal distance of 12.9 cm and a vertical distance of 9 cm relative to the first object (see Figure 1).

The starting position was marked with a round pole which participants had to hold. The large starting pole, used only in Experiment 1, had a height of 10.9 cm and a diameter of 3 mm and participants were instructed to grasp the starting pole where they could comfortably hold it. The small starting pole, used in Experiments 2 and 3, had a size of 4.5 cm and 3 cm respectively, also with diameters of 3 mm. Participants were instructed to grasp the pole at the very top, so that they would not have to move around it during grasping. When participants held the starting pole, they had to push down a button with the side of their hand. The release of this button sent a signal to the computer indicating the start of the movement. Participants always had to grasp the near object using index finger and thumb. After grasping it, they had to hold it up for a short time and then place it down on the table again. When estimating the size, they had to report whether the object in front was larger or smaller than the one in the back by saying “größer” (German for larger) or “kleiner” (German for smaller).

In Experiment 1 hand movements were recorded using an acoustic 3D movement registration system (Fa. Zebris, Tuebingen, Germany) with a sampling rate of 50 Hz. Two circular markers were attached to the most right lateral part of the thumb nail and the most left lateral part of the index finger nail, having the midpoint of the markers also be the midpoint of the nail-finger border. The markers were attached with medical tape. The cables connecting the markers to the Zebris system were attached to their upper arms giving them complete freedom of movement. In Experiments 2 and 3, we used the Vicon Motion Tracking System with Bonita Cameras and a sampling frequency of 100 Hz. This infrared optical 3D motion tracking system uses passive reflecting round markers, two of which were positioned, the same as in Experiment 1.

The aspects in which the experiments differed will be explained in the following sections.

### 2.2. Experimental Procedure

#### 2.2.1. Experiment 1

In this experiment, half of the participants first had to grasp the object closest to them and the other half first had to indicate whether the object was larger or smaller than the object placed further away from them. The white target objects were placed on a black surface. Participants were asked to close their eyes, grasp the starting pole and wait until they heard a sound indicating the start of the trial. They then had to open their eyes and begin with the first task. After 2.5 s, another two sounds were heard in quick succession, indicating the start of the second task. After another 2.5 s, a quick sequence of two tones indicated the end of the trial. The participants had to close their eyes again and wait until the next trial.

Object sizes and object positions were counterbalanced and randomized across trials. Participants performed a total of 96 trials. After blocks of 32 trials, participants could have a break. The experimental session started with 12 practice trials.

#### 2.2.2. Experiment 2

In Experiment 1, we did not replicate the original results reported by Ganel, Freud, Chajut, and Algom [30]. We therefore decided to perform another experiment to determine if we could replicate our own findings in a new sample of participants. Unfortunately, after we had already collected data of 10 participants (mean age = 28, range 20–40, 3 male), we noted that the large starting pole used in this experiment forced participants to first circumvent the top half of the starting pole before they could move their hand towards the target object. This resulted in a quite unusual grasping trajectory (see [37] for similar observations) and prompted us to tweak that aspect of our experiment by shortening the length of the starting pole. Consequently, we did not include the data of those first ten participants in our analyses and instead opted for recruiting 30 new participants. The experimental procedures in Experiment 2 were nearly identical to those employed in Experiment 1. The only differences were that the starting pole was shortened (as described above) and that participants now wore shutter glasses that controlled their vision.

#### 2.2.3. Experiment 3

In this last experiment we compared a closed-loop (full vision) condition, used also in Experiments 1 and 2, with an open-loop vision condition where vision was occluded during grasping. We wondered whether in the closed-loop condition, the potential influence of the perceived size might be reduced in its effect on MGA due to the availability of visual feedback (i.e., due to the fact that participants could observe their grasping hand and compare its aperture to the diameter of the target object, see Glover and Dixon [38] for a similar argument). To address this question, we introduced a condition where visual feedback was withdrawn at the start of the movement. To do so, we used an LCD shutter window which, when switched to its opaque status, occluded participants’ view of the target object and their own hand. In the closed loop condition, the shutter window turned transparent at the beginning of the trial and remained transparent until participants had completed their grasping movement. White objects were placed on a white surface. This ensured that the objects were clearly visible when the shutter window was transparent but were invisible when the window was switched to opaque. As we found in Experiments 1 and 2 that the task order was irrelevant, participants now always started with reporting the target’s size before grasping it. In all other respects the general procedure was identical to that employed in Experiment 1 and 2. In the open loop condition, the shutter window switched to transparent (open) at the beginning of the trial to allow participants to view the target objects. The window switched to opaque as soon as participants released the start button.

There were a total of 144 trials divided into 6 blocks with 24 trials per block. Size and position of the objects were randomized within the blocks. There were three closed loop blocks and three open loop blocks. For each participant, a new randomized sequence of closed and open-loop blocks was used. At the beginning of the experiment, participants were given 18 practice trials. After every two blocks, the participants were offered a break.

### 2.3. Participants

Participants in all experiments were right-handed by self-report and had normal or corrected-to-normal vision. Written consent of all participants was obtained prior to the studies. All experiments complied with the Code of Ethics of the World Medical Association Declaration of Helsinki [39] and were approved by the ethics committee of the University of Erlangen (Re.-No. 91_12 B). Participants were reimbursed with course credit or 8€ per hour. Each experiment lasted about 1.5 h.

#### 2.3.1. Experiment 1

Thirty participants (18 female) were tested for this experiment with an average age of 27 years (range 19–48). One participant had to be excluded due to technical problems with the motion tracker. Four further participants had to be excluded from the ANOVA analysis who had insufficient data in one of the data cells (e.g., one participant never judged a small object erroneously to be large in the third experimental block).

#### 2.3.2. Experiment 2

Thirty participants (16 female) were tested for this experiment with an average age of 27 years (range 19–53). In four participants, the kinematic data was corrupted and the participants had to be excluded. Five additional participants had to be excluded from the ANOVA analysis due to insufficient data in one of the data cells.

#### 2.3.3. Experiment 3

Thirty-three participants were tested with a mean age of 26 years (range 18–35). In three participants, the kinematic data was corrupted and the participants had to be excluded. Again, a further two participants had to be excluded from the ANOVA analysis since they had insufficient data in one of the cells. Of the remaining 28 participants, 17 were female.

### 2.4. Data Analysis

Ganel Freud, Chajut, and Algom [30] calculated the means of the MGAs for every participant and compared these means employing an ANOVA using *experimental block*, *object size*, and *verbal report* as within-subject variables. The variable *experimental block* indicates from which of the three experimental blocks the data originates, the variable *object size* denotes whether the target object was the smaller or the bigger on, and the variable *verbal report* (called *perceptual judgement accuracy* in the original report) indicates whether the data comes from the set of trials where the verbal judgement was correct (i.e., a small object was identified as small or the bigger object identified as the bigger one) or from the set of trials where the judgement was incorrect (e.g., the smaller object identified as the bigger one). In Reference [30] they found a significant main effect of *object size* that was, however, independent of the verbal report relating to the object’s size. All other effects were not significant. We analyzed the data the same way in Experiments 1 and 2 and without the variable experimental block in Experiment 3, since we found no effect of experimental block in Experiments 1 and 2.

We also carried out an additional analysis to examine whether the participants’ visuomotor system was truly better in discriminating between small and bigger objects than the participants’ perceptual system. To obtain measures of discrimination or classification accuracy on the basis of the MGA data we used an approach that has been employed in two previous studies [40,41]. The aim of this analysis was to use MGA values to determine whether the associated grasping movement was directed towards the smaller or the bigger object. In order to do so, we first had to decide on a cut-off value that best separates the MGAs for the small object and the big object. For any MGA values above the cut-off, one would assume that the target object was the bigger one, for any MGA values below the cut-off, one would guess that the target object was the smaller one. Clearly, some cut-off values are better than others in the sense that they produce more correct assignments. We decided to use the best cut-off value possible to give the claim by Ganel, Freud, Chajut, and Algom [30] the highest chance to be confirmed. In other words, we used the cut-off value that maximized the number of correct choices. We will call this measure optimal cut-off proportion or OC%.

For the subsequent ANOVA grasping trials were separated based on the size of the object, the experimental block, and based on the accuracy of the observer’s judgement. This caused the issue that in some cases data cells remained empty when, for example, a participant never perceived the small object as big. As the OC% analysis could still be computed, participants with empty data cells were excluded from the ANOVA but not from the OC% analysis.

All data is publicly available and can be accessed at www.zenodo.org, doi: 10.5281/zenodo.2577955.

## 3. Results

### 3.1. Maximum Grip Apertures (MGA) Analysis

#### 3.1.1. Experiment 1

**Figure 2:**
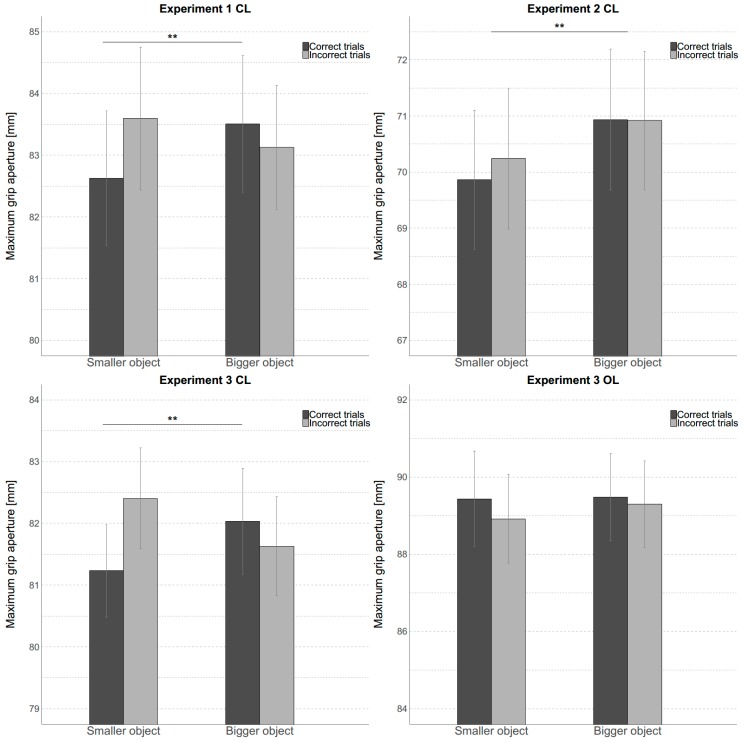
**MGA Analysis** Results of the Maximum Grip Apertures (MGA) analysis for the three experiments. CL: Closed Loop, OL: Open Loop. Error bars indicate one standard error of the mean. In Experiments 1 and 3 CL interaction effects between *object size* and *verbal report* were significant with pairwise comparisons showing that when participants judged correctly they grasped the small object with a smaller MGA and the large object with a larger MGA on average. In Experiment 2 there was a main effect of *object size*. No main or interaction effects were significant for Experiment 3 OL. ** indicates *p* < 0.01.

The results of the repeated-measures ANOVA with the factors *experimental block*, *object size*, and *verbal report* are shown in Figure 2 (upper left panel). We found that the only significant effect was the interaction effect between object size and *verbal report* (F(1,24) = 11.799, *p* < 0.01, η_p_^2^ = 0.33). Bonferroni corrected pairwise comparisons showed that MGAs for small and large objects differed significantly when participants’ judgement of the object’s size was correct (*p* < 0.001; small object: mean = 82.6 mm, CI [0.95] = 80.4 mm, 84.9 mm, versus large object: mean = 83.5 mm, CI [0.95] = 81.2 mm, 85.8 mm), but not when it was incorrect. Put differently, participants’ perceptual judgement about the size of the target object determined, at least to some extent, whether their grip aperture was correctly adjusted to the object’s size.

#### 3.1.2. Experiment 2

The results were analyzed identically to Experiment 1 and are shown in Figure 2 (upper right panel). In this experiment, we replicated the results of Ganel, Freud, Chajut and Algom [30]. The only significant effect was the main effect of *object size* (F(1,20) = 11.954, *p* < 0.01, η_p_^2^ =0.374), with MGAs being smaller (mean = 70.1 mm, CI [0.95] = 67.5 mm, 72.6 mm) when the small object was the target and being bigger (mean = 70.9 mm, CI [0.95] = 68.4 mm, 73.5 mm) when the big object was the target, independent of the correctness of the perceptual judgement (i.e., no interaction effect).

#### 3.1.3. Experiment 3

We calculated a repeated-measures ANOVA using *vision* (open-loop versus closed-loop), *object size*, and *verbal report* as within-subject variables. The results are shown in Figure 2 (lower panels). We found a main effect of *vision*, (F(1,27) = 81.751, *p* < 0.001, η_p_^2^ = 0.752), a two-way interaction effect between *vision* and *verbal report* (F(1,27) = 4.828, *p* = 0.037, η_p_^2^ = 0.152), and a three-way interaction effect between *vision*, *object size*, and *verbal report* (F(1,27) = 5.479, *p* = 0.027, η_p_^2^ = 0.169).

To follow-up the 3-way interaction effect, we calculated two separate repeated-measures ANOVAs for each vision condition separately with the factors *object size* and *verbal report*. For the closed loop condition, we found a significant interaction effect between *object size* and *verbal report* (F(1,27) = 12.781, *p* < 0.01, η_p_^2^= 0.321). Bonferroni corrected pairwise comparisons showed that the difference in MGA when grasping larger and smaller objects was significant only when participants judged their sizes correctly (*p* < 0.01). That is, participants showed smaller MGAs when the small object was presented which they correctly judged to be small (mean = 81.2 mm, CI [0.95] = 79.7 mm, 82.8 mm) and larger MGAs when the larger object was grasped which they correctly judged as being larger (mean = 82.0 mm, CI [0.95] = 80.3 mm, 83.8 mm). When participants incorrectly judged the object size the resulting difference in MGA between small and large object just failed to reach significance (*p* = 0.058). The same ANOVA for the open-loop vision condition revealed no significant main or interaction effects suggesting that MGAs were unaffected by any of the experimental variations.

To sum up, the findings obtained for Experiment 3 in the closed-loop condition closely resembled those obtained in Experiment 1. In particular, we found that perceived size significantly influenced the MGAs.

### 3.2. Cut-Off Based Analysis

#### 3.2.1. Experiment 1

Ganel, Freud, Chajut, and Algom [30] argued that the visual information available to the visuomotor system guiding the grasping movements is more accurate than the information available to the perceptual system on which the verbal reports of participants is presumably based. To directly test this claim, we compared participants’ verbal accuracy with their motor accuracy. The verbal accuracy (i.e., the percentage of trials where participants correctly called the small object small and the bigger object big) was 69%. This percentage is higher than the verbal accuracy reported by Ganel, Freud, Chajut, and Algom [30] but within the conventional range of uncertainty for psychophysical measures (25%–75%). To obtain a measure of how accurately the grasping performance predicted the real size of the target object, we used the procedure described above (General methods; data analysis).

**Figure 3:**
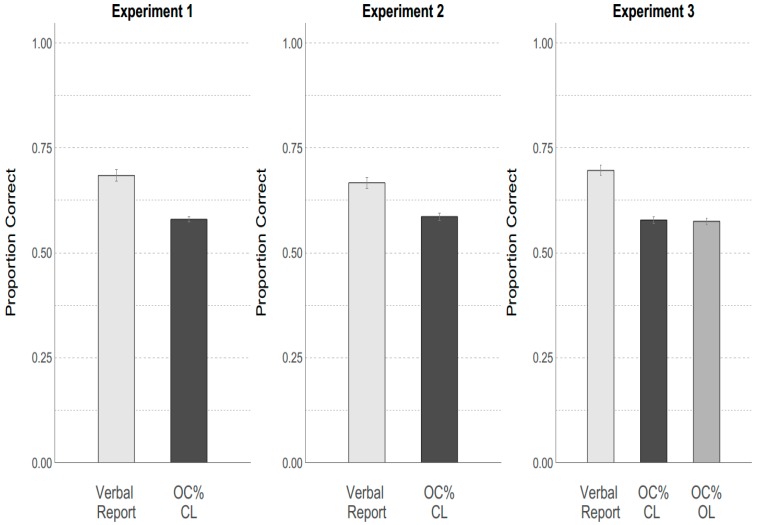
**Proportion correct values for Experiments 1–3.** Size-classification was consistently superior when based on participants’ verbal reports than when based on participants’ grasping performance (OC%: optimal cut-off proportion). Notably this was also the case in Experiment 2, where we directly replicated the findings of Ganel, Freud, Chajut, and Algom [30]. Error bars indicate one standard error of the mean. CL: Closed Loop, OL: Open Loop.

We then compared verbal accuracy with motor accuracy (based on the threshold-technique) using paired-samples *t*-tests. The results are shown in Figure 3 (left panel). A Shapiro–Wilk test confirmed that the sampling distribution was normally distributed. The difference was highly significant with the mean of the perceptual task being higher (mean = 0.69, SD = 0.076) than the mean of the criterion-based values, OC% (mean = 0.58, SD = 0.032; *t*(28) = 7.408, *p* < 0.001). The findings suggest that grasping performance predicts the correct target size less reliably than the participants’ verbal report. Thus, if there is a difference in visual-size related information quality for the visuomotor (dorsal) and perceptual (ventral) system, the difference is exactly opposite to the one claimed by Ganel, Freud, Chajut, and Algom [30].

#### 3.2.2. Experiment 2

As in Experiment 1, we calculated the OC%. The results are shown in Figure 3 (middle panel). The average percentage of correct visual identifications in this experiment was 66.7%. Again, the sampling distribution was not significantly different from normal as assessed by a Shapiro–Wilk test. We then calculated a paired-samples t-test comparing the percentage of correct trials as reported by the participants with OC%. The difference was highly significant with participants again being more accurate in their verbal report values (mean = 0.67, SD = 0.068) than in the OC% values (mean = 0.59, SD = 0.044; *t*(25) = 4.942, *p* < 0.001).

To sum up, we found a clear effect of object size on MGA. This effect was not modulated by the perceived size, i.e., independent of the observer’s verbal judgement. Nevertheless, OC% again confirmed as before (see Experiment 1) that classification accuracy based on MGA is in fact worse than that obtained for the verbal report.

#### 3.2.3. Experiment 3

We used the same measure as described before to estimate classification accuracy (small versus larger object) for the verbal and motor measures, but this time we calculated this measure separately for open- and closed-loop vision conditions. The results are shown in Figure 3 (right panel). The sampling distributions were not significantly different from normal as assessed with a Shapiro–Wilk Test. In the closed-loop condition, the percentage of correct trials in the perceptual condition (mean = 0.70, SD = 0.066) was significantly larger than the percentage of correct values as assessed by criterion values, OC%, (mean = 0.58, SD = 0.42; *t*(29) = 7.810, *p* < 0.001). In the open-loop condition the results were similar, with the perceptual identification of object size (mean = 0.70, SD = 0.066) being significantly larger than the OC% values (mean = 0.58, SD = 0.042; *t*(29) = 9.072, *p* < 0.001). Thus, as before, our findings suggest that, if anything, the classification based on the participants’ verbal report is better than classification based on their grasping.

## 4. Discussion

Ganel, Freud, Chajut, and Algom [30] presented participants with two very similarly sized target objects. The diameter of the smaller object was just 0.5 mm less than that of the bigger object. When asked to tell whether a given object was the smaller or the bigger one, their performance was at chance level. However, when asked to grasp one of the two objects, the average grip aperture for the smaller object was significantly smaller than that found for the bigger object. It was concluded that the visual information available for motor performance is more precise than that available to conscious perception – which has been presented as a further piece of evidence in favor of the hypothesis that vision for action and perception are based on distinct visual representations and are served by different anatomical systems [1,2]. In a series of three experiments, we aimed to replicate and extend the results presented by Ganel, Freud, Chajut, and Algom [30]. We aimed to answer two questions. Firstly, can we replicate the original results, i.e., the finding that participants’ grasping movements distinguish between object sizes that participants are unable to discriminate verbally. The second, more important, question was, however, whether such a difference really implies that the visual information available to the visuomotor system is more precise than the information available to the perceptual system. Such a claim would lead us to expect that the classification of objects into smaller and bigger objects can be done more reliably on the basis of measures derived from motor performance than from verbal performance.

Let us look at the first question of whether we can replicate the main finding from the Ganel, Freud, Chajut, and Algom [30] study. The answer is no, not consistently. Ganel, Freud, Chajut, and Algom [30] found that MGA was determined by the actual size of the object irrespective of whether participants identified the size of the object correctly or not. In contrast, we found that in two out of three experiments, participants’ beliefs about the object’s size influenced their MGAs. What about the second claim that the precision of the size-information reflected in the grasping performance is superior to the size-information used for the perceptual report? We found consistently (in all three experiments) the opposite pattern. When using classification accuracy, we found that motor-based classification is worse than classification based on participants’ verbal reports. Taken together, the findings from our three experiments suggest that visual information used for grasping is not better than information available to the perceptual system. While Ganel, Freud, Chajut, and Algom [30] used their findings to support the PAM our analysis suggests that their results do not actually demonstrate the superiority of visuomotor classification as compared to perceptual classification.

In this context it is interesting to note that in the second experiment, where our data followed the same pattern as observed by Ganel and colleagues, the re-analysis of the data using the motor-classification approach nevertheless showed that classification based on perception is better than classification based on action. This demonstrates the potential of the motor-classification approach to allow a direct comparison of information used in motor tasks and non-motor tasks. It also means that our study coming to a different conclusion than Ganel and colleague’s is primarily due to our use of a different type of analysis and does not just present a failure to replicate.

In the following we will explore the implications of our findings and discuss some open questions and limitations of our study.

### 4.1. The Role of Knowledge in Visuomotor Performance

The role of knowledge in visuomotor performance is a contentious issue. According to the division of labor within the visual system as suggested by Milner, Goodale, and colleagues [1,2], vision in the ventral system is used to identify objects. Object identification allows the cognitive system to retrieve stored information about an object. The dorsal system, in charge of using vision for guiding actions, does not have direct access to such memorized information. Consequently, visuomotor responses are expected to remain unaffected by our knowledge about objects. This prediction was tested by McIntosh and Lashley [36] and found to be wrong. When participants were presented with objects whose actual size did not correspond to their familiar and expected size, they made errors in their grasping responses. This demonstrates that participants’ knowledge determines, at least in part, their visuomotor response. Similar findings were obtained by others [41,42,43,44]. Ganel, Freud, Chajut, and Algom’s [30] study addressed a similar question in a somewhat different way. Presenting target objects that appeared to the observer to be near-identical led to numerous instances where the observer’s judgement about the object’s size and its true size were at odds. This provided an opportunity to test whether it is the perceived size or the actual size of the object that drives the grasping response. Ganel, Freud, Chajut, and Algom [30] argued that the perceived size was irrelevant and only the actual size affected the grip aperture. However, our findings are in conflict with this conclusion. Two out of three experiments demonstrated a significant influence of the perceived size on grasping. On the basis of our findings, we conclude that beliefs or knowledge about objects affect object-oriented actions, a finding that undermines the hypothesis that the visual processes in the ventral and dorsal systems are independent of each other.

### 4.2. Classification Versus Comparing Means

Our results show that the type of analysis used will determine what conclusions are drawn from the data. Ganel, Freud, Chajut, and Algom [30] focused on the significant MGA difference between trials with small versus big objects and concluded that the grasping hand can distinguish more reliably between small and big objects than the perceptual system of the observer. More importantly, however, they did not test how good classification performance would actually be, were they to use MGAs to guess whether the target was the big or the small object. Here, we computed this classification performance and found that classification based on the grip aperture size is less reliable than classification based on observers’ verbal reports.

It is easy to be fooled into the belief that finding a significant difference indicates above chance discrimination performance. This potential fallacy is not restricted to the field of motor control. Franz and von Luxburg [45] recently demonstrated that the same problem also occurs in a very different cognitive domain. They re-examined data from a study on lie-detection [46]. ten Brinke, Stimson, and Carney [46] asked volunteers to judge whether people on videos were lying or telling the truth (i.e., dichotomous measure). It turned out that most observers were poor at that task and their guesses were hardly better than chance. However, the researchers speculated that observers’ *implicit* ability to distinguish between liars and truth-tellers might be superior. They tested this idea in a priming experiment. Static images of liars and non-liars were presented shortly before words relating to lying or truthfulness were flashed onto the screen. Observers had to classify those words and it was found that response times (i.e., continuous measure) were faster whenever the meaning of the word and the truthfulness of the presented face were congruent, i.e., observers were quicker to classify a word related to lying when this word was preceded by a face belonging to a liar than when it was preceded by a face belonging to an honest person. Franz and von Luxburg [45] took the response times and applied various classification procedures. They found that the accuracy of the classification based on the response times in the priming task was no better than the classification accuracy based on observers’ verbal responses. This example and our own findings in this study illustrate why we should be wary of any dissociation that contrasts a significant mean difference of a continuous measure in one task with a non-significant classification ability in a different task.

### 4.3. Is Size-Information for Motor Control Inferior to Size-Information in the Perceptual System? 

We did not just find that size-classification based on motor performance is not better than perceptual size-classification. In fact, we found that motor-based size-classification is worse. One might argue that this also implies the existence of distinct representations of visual size in dorsal and ventral pathways. Thus, proponents of the perception-action model might be tempted to conclude that, while our findings may prompt some minor adjustments to the model, this finding still provides support for the PAM’s core claim of distinct visual representations for perception and action. However, this conclusion assumes that the trial-by-trial distribution of grip aperture values is entirely driven and shaped by the trial-by-trial distribution of the represented sizes in the visual system. This assumption is, however, implausible. The value of the grip aperture in any given trial is likely to be the result of many factors: the estimated size of the object, the current noise in the motor system, and the intended approach angle to name just a few possible factors that can influence the maximum grip aperture. As we have argued elsewhere [23,24,32], it is in our view a mistake to treat the MGA as a perfect read-out of the motor system’s estimate of the target object’s size. Other constraints and sources of noise will contaminate this measure. Given these considerations, it should perhaps not surprise that classification based on grasping is worse than classification based on verbal report. In addition, it is important to point out that we also found a direct influence of perceptual judgement on grip aperture in two of our experiments. This finding suggests that the visual information used in perception is also used in action.

### 4.4. Manual Estimation

Someone familiar with the original study by Ganel, Freud, Chajut, and Algom [30] might wonder why we did not use a so-called manual-estimation (ME) condition in our study and whether this limits or compromises the conclusions we can draw from our findings in any way. In the ME condition of the original study participants were asked to indicate the size of the target object using their index finger and thumb. Their results for the manual-estimation condition followed the findings for the perceptual (verbal report) task. However, there are several reasons why we think the absence of a manual estimation condition is not a critical issue for our study and does not affect its main conclusions.

Firstly, it should be noted that Ganel and colleagues primarily based their conclusions on the findings from their verbal report and not on those from the ME condition. For example, when they emphasize that the hand seems to distinguish between objects that are perceptually indiscriminable, they refer to a perceptual threshold based on observers’ verbal report. Furthermore, the key statistical finding reported and used in support of the PAM is the significant difference in hand-opening between the two differently sized objects that is found irrespective of the observers’ verbal judgements. In contrast, the findings from the ME condition are only compared in a qualitative manner to those obtained in the grasping condition. It is remarked that while ME mirrors observers’ verbal assessments, the same is not true for the grasping condition. However, no statistical comparison between the ME condition and the grasping condition is reported, and thus we do not know whether this difference is reliable (for a more detailed discussion of this problem, see [47]).

Secondly, the absence of the ME condition does not affect our main argument, which is that comparisons between continuous and dichotomous performance measures can be highly misleading. We demonstrated that even if statistical tests suggest that the task with the continuous measure is more sensitive than the task with the dichotomous measure, a comparison of the two tasks based on a mapping of both measures onto a dichotomous level can negate, or even contradict, this conclusion. Given that such comparisons are frequently used in the discussion of the PAM, our conclusions remain valid and relevant to this specific scientific debate regardless of whether or not a ME condition is included.

Thirdly, a little thought experiment illustrates that the addition of a ME condition would not substantially affect the implications of our study. We demonstrated that grasping when probed for its ability to discriminate between the small and the big object does no better job than observers’ verbal report. Now, let us assume we added a ME condition and analyzed it the same way as the grasping condition. Two outcomes are possible: It turns out that discrimination is not worse or potentially even better for ME than for grasping. In this case the findings from ME provide further support for our conclusion that accuracy of vision for perception is not worse than accuracy of vision for action. Alternatively, we might find that ME is in fact worse than grasping (as would be hypothesized by the proponents of the PAM). In this case, we would have conflicting results. One measure for perception shows accuracy that is superior to grasping, while the other perceptual measure suggests that accuracy is inferior to grasping. What would we learn from this result? Most likely, we would conclude that grasping is not in general better than perception (but that it depends on the exact perceptual measure used) and that thus one could not use the findings to support the PAM (for examples of such data constellations, see [48]).

Thus, if someone intended to derive PAM-supporting evidence from such a data-pattern, they would have to resort to the assumption that ME is a truer measure of perception than verbal reports. We think such an assumption is not necessarily justified. As we have argued before [24,33], ME is not an unproblematic task. Its direct comparison with grasping is made difficult by confounds. In grasping, participants can use direct visual and haptic feedback to improve their performance over time. The same is not true for ME where they receive either no, or only indirect or delayed visual and haptic feedback. As we have reported elsewhere [49], feedback where the endpoint of the action cannot be directly related to the target positions is not very useful.

To conclude, our study refutes the claim by Ganel, Freud, Chajut, and Algom [30] that the visuomotor system can discriminate between object sizes more reliably than the perceptual system. In fact, our results suggest that the opposite is true. Moreover, we found that the perceptual judgement has a direct effect on the grasping performance. Taken together, our findings further undermine the claim of distinct representations of visual size for perception and action.
