# Correction: Göhringer et al. Grasping Discriminates Between Object Sizes Less Not More Accurately than the Perceptual System. *Vision* 2019, *3*, 36

**DOI:** 10.3390/vision10040058

**Published:** 2026-08-24

**Authors:** Frederic Göhringer, Miriam Löhr-Limpens, Constanze Hesse, Thomas Schenk

**Affiliations:** 1 Lehrstuhl für Klinische Neuropsychologie, Ludwig-Maximilian University Munich, Leopoldstr. 13, 80802 Munich, Germany; miriam.loehr@psy.lmu.de (M.L.-L.); thomas.schenk@psy.lmu.de (T.S.); 2 School of Psychology, University of Aberdeen King’s College, William Guild Building, Aberdeen AB24 3FX, UK; c.hesse@abdn.ac.uk

There was an error in the original publication [1]. Two of the data analyses in Sections 3.1.2 and 3.1.3 contained some errors that were due to the mislabeling of data used in the analyses. In the following we present the corrected results and the corrected figure (Figure 2). These changes do not affect the conclusions of the article, since these conclusions are not based on the results of these analyses.

For Experiment 2 (Section 3.1.2) the main effect of *object size* is F(1,20) = 9.247, *p* < 0.01, η_p_^2^ = 0.316, with MGAs being smaller (mean = 70.1 mm, CI [0.95] = 67.5 mm, 72.6 mm) when the small object was the target and being bigger (mean = 70.9 mm, CI [0.95] = 68.4 mm, 73.3 mm) when the big object was the target, independent of the correctness of the perceptual judgment (i.e., no interaction effect).

For Experiment 3 (Section 3.1.3) there was no three-way interaction effect, but a two-way interaction effect between *object size* and *verbal report*, F(1,27) = 9.412, *p* < 0.01, η_p_^2^ = 0.258. Bonferroni corrected pairwise comparisons showed that the difference in MGA when grasping larger and smaller objects was significant only when participants judged their sizes correctly (*p* < 0.01). That is, participants showed smaller MGAs when the small object was presented which they correctly judged to be small (mean = 85.1 mm, CI [0.95] = 83.3 mm, 86.9 mm) and larger MGAs when the larger object was grasped which they correctly judged as being larger (mean = 85.8 mm, CI [0.95] = 83.9 mm, 87.7 mm). Furthermore, the difference between MGAs was only significant between correctly and incorrectly judged sizes for the small object. That is, participants showed smaller MGAs when the small object was presented and they correctly judged it to be small (mean = 85.1 mm, CI [0.95] = 83.3 mm, 86.9 mm) and larger MGAs when the small object was presented and they incorrectly judged it to be larger (mean = 85.9 mm, CI [0.95] = 84.0 mm, 87.8 mm). The results in Experiment 3 thus more closely resemble the results in Experiment 1 than the original analysis suggested.

Due to this there was also a mistake in Figure 2. The figures for Experiment 2 CL and Experiment 3 OL were incorrect. In Experiment 2 CL the value for the condition when the object was bigger and the verbal report was correct was lower than reported. In Experiment 3 OL the values for the smaller object were switched. The corrected Figure 2 appears below with an updated legend.

**Figure 2:**
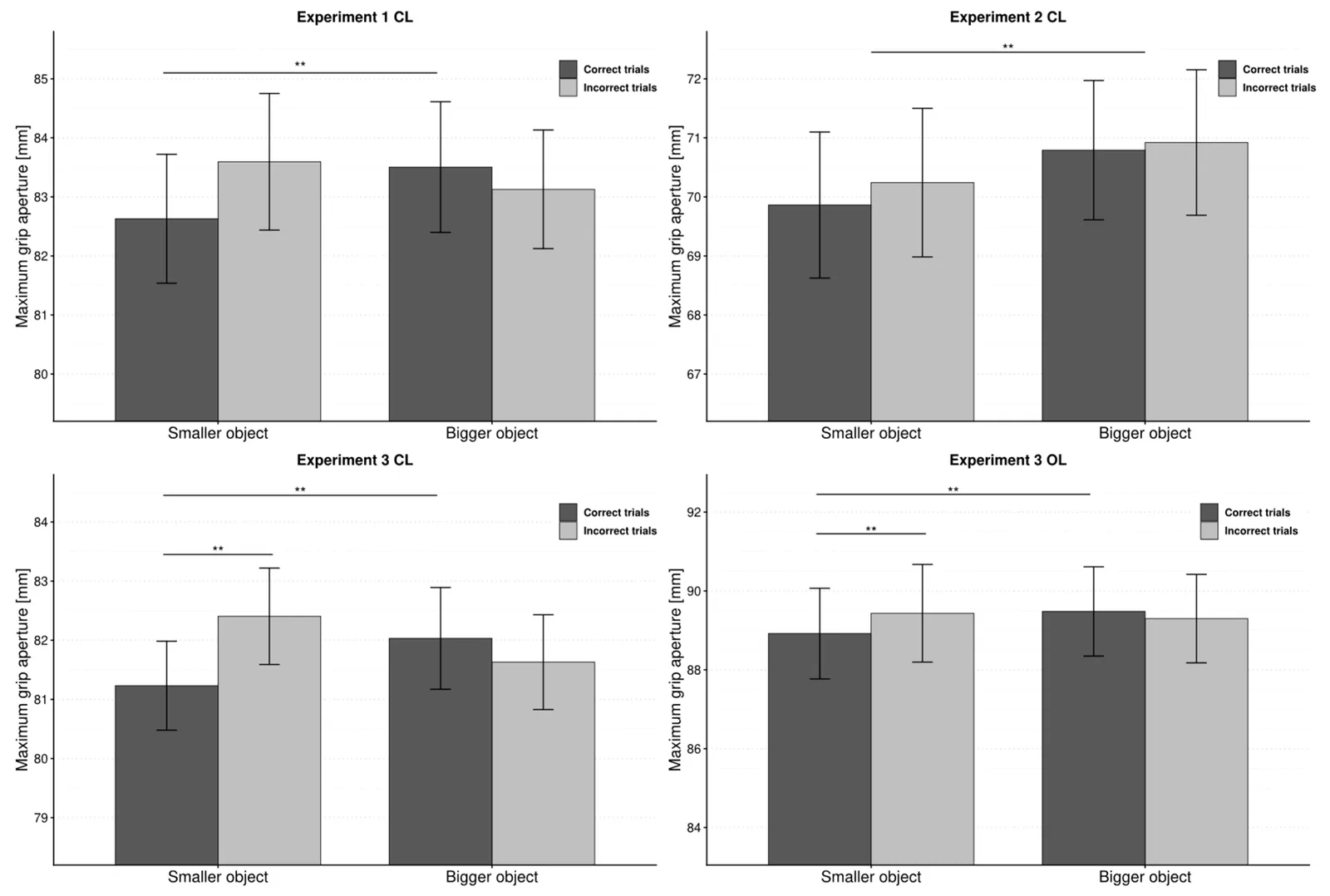
Results of the maximum grip apertures (MGA) analysis for the three experiments. CL: Closed Loop, OL: Open Loop. Error bars indicate one standard error of the mean. In Experiments 1 and 3 interaction effects between *object size* and *verbal report* were significant with pairwise comparisons showing that when participants judged correctly, they grasped the small object with a smaller MGA and the large object with a larger MGA on average. In Experiment 2 there was a main effect of *object size*. Please note that in Experiment 3, the 3-way interaction involving the comparison between CL and OL was not significant. Accordingly, the CL and OL condition were analyzed jointly. This means that the significant difference between correct and incorrect trials for the small objects and the significant difference between small and big object for correct trials, indicated by ** reflect the results from an analysis based on the dataset that combined trials from CL and OL. ** indicates *p* < 0.01.

The authors state that the scientific conclusions are unaffected. This correction was approved by the Academic Editor. The original publication has also been updated.
